# Global loss of acetylcholinesterase activity with mitochondrial complexes inhibition and inflammation in brain of hypercholesterolemic mice

**DOI:** 10.1038/s41598-017-17911-z

**Published:** 2017-12-20

**Authors:** Rajib Paul, Anupom Borah

**Affiliations:** 1 Cellular and Molecular Neurobiology Laboratory, Department of Life Science and Bioinformatics, Assam University, Silchar, 788011 Assam India; 2 Department of Zoology, Pandit Deendayal Upadhyaya Adarsha Mahavidyalaya (PDUAM), Eraligool-788723, Karimganj, Assam India

## Abstract

There exists an intricate relationship between hypercholesterolemia (elevated plasma cholesterol) and brain functions. The present study aims to understand the impact of hypercholesterolemia on pathological consequences in mouse brain. A chronic mouse model of hypercholesterolemia was induced by giving high-cholesterol diet for 12 weeks. The hypercholesterolemic mice developed cognitive impairment as evident from object recognition memory test. Cholesterol accumulation was observed in four discrete brain regions, such as cortex, striatum, hippocampus and substantia nigra along with significantly damaged blood-brain barrier by hypercholesterolemia. The crucial finding is the loss of acetylcholinesterase activity with mitochondrial dysfunction globally in the brain of hypercholesterolemic mice, which is related to the levels of cholesterol. Moreover, the levels of hydroxyl radical were elevated in the regions of brain where the activity of mitochondrial complexes was found to be reduced. Intriguingly, elevations of inflammatory stress markers in the cholesterol-rich brain regions were observed. As cognitive impairment, diminished brain acetylcholinesterase activity, mitochondrial dysfunctions, and inflammation are the prima facie pathologies of neurodegenerative diseases, the findings impose hypercholesterolemia as potential risk factor towards brain dysfunction.

## Introduction

Acetylcholinesterase (AChE) is an enzyme of brain cholinergic system that hydrolyses the neurotransmitter acetylcholine to choline and acetate in the synaptic cleft^1,2^. Mounting evidence has shown reduced activity of AChE in several brain disorders, including neurodegenerative disorders^3–9^. Mainly, loss of AChE is evident in forebrain of Alzheimer’s disease (AD) patients, which are revealed from Positron emission tomography and autopsy studies^5–9^. In addition to brain, reduced activity of AChE was also found in cerebrospinal fluid, plasma, erythrocytes and lymphocytes of AD patients as compared to age-matched subjects^10–12^.

Escalating evidence has depicted that elevated cholesterol level in blood plasma (hypercholesterolemia) is a prognostic risk factor for neurodegenerative diseases, including AD^13–15^. Epidemiological as well as experimental model studies convincingly demonstrated the appearance of cognitive impairment and dementia in hypercholesterolemic condition^14,16–24^. Impairment of cholinergic neuronal system in brain was reported to be the underlying event of cognitive impairment in hypercholesterolemic rat^16^. Thereby, hypercholesterolemia has been brought into the domain of risk factors for AD. Although hypercholesterolemia is linked with AD, and loss of AChE is an early event of the disease, studies in animal models have provided inconsistent results regarding the effect of hypercholesterolemia on brain AChE activity^22–25^. Meanwhile, mitochondrial dysfunction at respiratory complexes and resulting oxidative stress reported in brain of hypercholesterolemic mice, which were however limited to cortical region^26,27^; while neuro-inflammatory stress was evident in cortex and hippocampus as well^14,16,28^. Moreover, cholesterol homeostasis in brain is regulated through *de novo* synthesis, with limited import from the peripheral circulation to the brain^29,30^ therefore, the effect of hypercholesterolemia on brain cholesterol levels is largely unknown. Here, we aimed to investigate the impact of hypercholesterolemia on the functional status of AChE and mitochondrial complexes, and inflammation in four discrete brain regions (cortex, striatum, hippocampus and substantia nigra), to unveil its influences on brain functions. We also tested if the elevated levels of cholesterol in blood have any influence on its level in brain.

## Materials and Methods

### Animals

Swiss Albino male mice of eight weeks old having body weight 20–22 g were used in the present study. The animals were procured from Pasteur Institute, Shillong India. The mice were housed under standard laboratory conditions of temperature (24 ± 2 °C), humidity (60 ± 5%) and 12 h light/dark cycles. During the study period, mice were kept individually in polypropylene cages (Tarsons, India) with free access to food and purified drinking water. The experimental protocols used in the present study have been approved by the Animal Ethics Committee, Assam University, Silchar, India (IEC/AUS/2013-052, dt-20/3/13). All methods were performed in accordance with the relevant guidelines and regulations.

### Chemicals

Cholesterol (97900), Evans Blue dye (EBD; 46650), 5,5′-dithiobis-(2-nitrobenzoic acid) (DTNB; 32363), Nicotinamide adenine dinucleotide (Reduced) disodium salt (NADH; 77268), sodium succinate (87578), nitroblue tetrazolium (NBT; 48898), cytochrome c (81551), 3,3-diaminobenzidine (DAB; 94524), sodium azide, Triton X-100 and other chemicals of extra-pure grade were purchased from SISCO Research Laboratories, India. Acetylthiocholine iodide (01480), coenzyme Q_0_, DAB liquid substrate kit (D3939), tissue cholesterol estimation kit (MAK043) and poly L-lysine were purchased from Sigma-Aldrich, USA. Primary antibody against mouse Glial-fibrillary acidic protein (GFAP; ab7260) raised in rabbit and donkey serum were purchased from Abcam, Cambridge, UK. Goat anti-rabbit secondary antibody tagged with horseradish peroxidase (HRP; AP307P) was purchased from Millipore Co., USA. Serum cholesterol estimation kit (CHOL, Autopak) was obtained from Siemens Ltd., India.

### Experimental design

**Figure 1:**
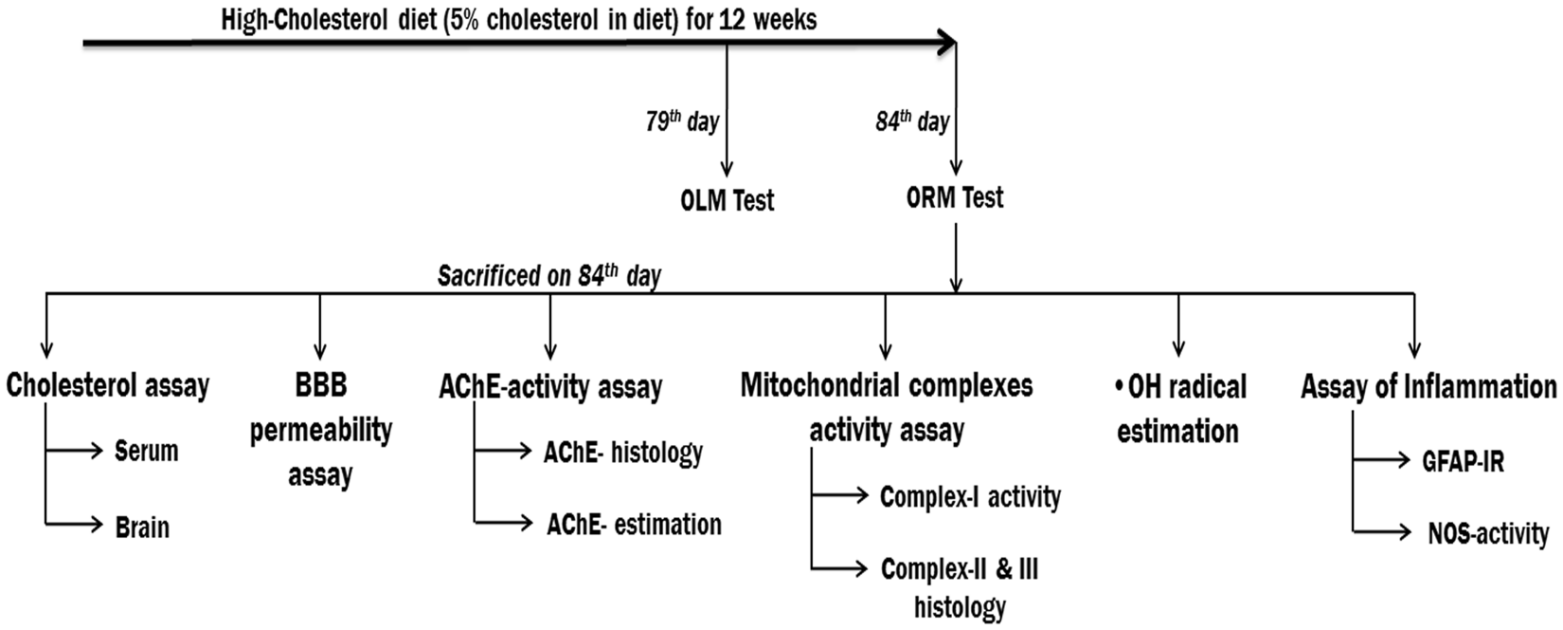
Timelines of the experiment. Abbreviations: OLM, object location memory; ORM, object recognition memory; BBB, blood-brain barrier; AChE, Acetylcholinesterase; •OH, hydroxyl radical; GFAF-IR; Glial fibrillary acidic protein-Immunoreactivity; NOS, nitric oxide synthase.

To induce hypercholesterolemia, mice were provided with high-cholesterol diet (HCD; 5% w/w cholesterol mixed with normal rodent chow) for 12 weeks *ad libitum*, which was established in our laboratory^31^. Mice were randomly divided into two groups based on their diets, such as control (provided normal diet) and HCD. Animals were acclimatized in each of the memory function test apparatus without any objects 24 h before the test day and then subjected to memory function using object location (for short-term memory) and object recognition (for long-term memory) tests on 79^th^ and 84^th^ day respectively and sacrificed on the last day of 12 weeks treatment (84^th^ day). Hypercholesterolemia in mice was established by measuring serum total cholesterol level. Cholesterol level, and activity of AChE and mitochondrial complex-I were estimated using spectrophotometer from four different regions of brain viz. cortex, striatum, hippocampus and midbrain (mainly substantia nigra region). A set of mice (n = 5/group) was used for determination of blood-brain barrier (BBB) permeability in whole brain lysate. For histoenzymological studies (activity assay of AChE, complex-II, III, and NOS-histology), the anesthetized mice were perfused with 10% glycerol, and for GFAP-immunoreactivity mice were perfused with 4% paraformaldehyde. Another set of mice (n = 5/group) were sacrificed 1 h post injection with salicylic acid on the last day of treatment for analysis of hydroxyl radical in different regions of brain. The timeline of different experiments was mentioned in Fig. 1.

### Cognitive function test

Object location memory (OLM) and object recognition memory (ORM) test of the experimental animals were performed to examine the role of hypercholesterolemia in memory^32^. The tests were performed following Vogel-Ciernia *et al*.^32^ with slight modifications. For performing OLM test, two identical objects were placed 7 cm apart from each other in a chamber of 30 cm × 23 cm dimensions. This rectangular chamber had 2 longer sides (30 cm each) and 2 shorter sides (23 cm each) opposite to each other respectively. The objects were placed 1 cm away from shorter sides and 6.5 cm away from longer sides of the chamber. Animals were placed on the opposite side of the object and 2.5 cm away from shorter wall of the chamber. Animals were trained in this setup for 10 min. After an interval of 90 min post-training, test was done in which one of the objects was shifted to a new location. The exploration time for both the objects was recorded for 5 min to calculate the discrimination index (DI).

ORM test was performed using a cylindrical chamber of 28 cm diameter. Objects were placed diagonally 8 cm apart from each other and 3 cm away from the circumference. On 4^th^ day following OLM test animals were trained for ORM with similar objects for 10 min each. After an interval of 24 h posttraining, ORM test was performed for 5 min with a novel object and the exploration time in both the object was recorded to calculate the DI.

### Blood and brain cholesterol estimation

Blood was collected by cardiac-puncture and serum was separated for estimation of cholesterol. Cholesterol was assayed by the enzymatic method using a colorimetric kit following the manufacturer’s instructions (CHOL, Autopak, Siemens)^31^.

Brain cholesterol level was estimated using a commercially available kit. Briefly, the animals were perfused transcardially with 30 ml phosphate-buffered saline (PBS, 0.1 M; pH 7.4) followed by removal of brain from calvarium. Half hemisphere was used for cholesterol estimation and the other half was used for estimation of AChE activity. The different regions of the brain were micropunched or separated out, lysed in 1% Triton X-100 in chloroform and centrifuged at 14,000 × g for 10 min. Organic phase was collected and dried at 50 °C for 30 min, followed by vacuum drying for 30 min to remove any trace of chloroform^33^. The dried lipids were re-suspended in assay buffer and used for quantification of cholesterol following manufacturer’s instructions using a Microplate Spectrophotometer (Multiskan GO, Thermo Fisher Scientific, Finland).

### Histochemical assay of brain cholesterol

Effect of hypercholesterolemia on brain cholesterol was assayed following Schultz’s method with minor modifications^34^. The method is based on the principle of the Liebermann-Burchard reaction, which gives blue or greenish-blue colour for cholesterol^35^. Twenty-micron thick sections from paraformaldehyde (4% w/v in PBS) fixed discrete regions of brain were incubated in aqueous solution of 2.5% (w/v) ferric ammonium sulfate at room temperature. After five days of incubation, sections were rinsed in distilled water and mounted on slides. A drop of freshly prepared mixture of glacial acetic acid and sulphuric acid (1:1) was poured over each section, coverslipped immediately and photographed using digital SLR camera attached to the microscope.

### Brain acetylcholinesterase activity

AChE activity was estimated from different regions of brain following Ellman *et al*.^36^. The different brain regions from each group were homogenized separately in Tris-HCl buffer (50 mM; pH 7.4) in 1:20 ratio (w/v). The reaction mixture contains 0.1 ml tissue homogenates and 0.8 ml of DTNB in 0.9 ml of Tris–HCl buffer. The reaction was initiated by addition of 0.2 ml of acetylthiocholine iodide. The absorbance of coloured complex was recorded at 412 nm for 1 min at 25 °C using Spectrophotometer. The protein concentration of tissue homogenates was determined using the Folin phenol reagent^37^.

### BBB permeability assay

BBB permeability assay was performed using Evans Blue dye (EBD) extravasation method of Qi *et al*.^38^ with slight modifications^39^. On the last day of treatment period, animals were injected with 2% EBD at the dose of 4 ml/kg body weight. After 22 h of injection, mice were sacrificed; perfused transcardially with 50 ml PBS (0.1 M; pH 7.4) and brains were dissected out. A 10% w/v homogenate of whole brain was prepared in 50% trichloroacetic acid and centrifuged at 12000 × g for 20 min. The supernatant was diluted with 95% ethanol (1:3 v/v), and the absorbance was measured at 620 nm using Spectrophotometer.

### Mitochondrial complex-I activity

Complex-I activity was assayed from the mitochondrial fractions of cortex, striatum, hippocampus and substantia nigra, according to Saravanan *et al*.^40^ with minor modifications previously reported by us^34^. Mitochondrial fractions were prepared from the intended brain regions of treated and control animals as described earlier^41^. Animals were killed by decapitation and the left and right brain hemisphere of the individual animal was homogenized together in 10 volumes of ice-cold potassium phosphate buffer (10 mmol/L; pH 7.2) containing 0.32 mol/L sucrose. The first pellet was discarded after centrifugation (1000 × g for 10 min at 4 °C) of the homogenate and the second pellet was obtained by centrifugation of the supernatant at 10,000 × g for 30 min at 4 °C. The pellet was resuspended in potassium phosphate buffer (10 mmol/L; pH 7.2) and used for enzyme assay. The 1 ml reaction mixture contained potassium phosphate buffer (10 mM; pH 7.2), sodium azide (5 mM); coenzyme Q_0_ (50 mM) and 90–110 mg mitochondrial fractions. After preincubation for 3 min at 32 °C, the reaction was initiated by the addition of NADH (120 mM) and the rate of decrease in the absorbance was monitored at 340 nm for 2 min.

### Hydroxyl radical estimation

For investigating the effect of hypercholesterolemia on generation of hydroxyl radical (•OH) in discrete brain regions, the adducts of salicylic acid, 2,3- and 2,5-DHBA, were estimated using HPLC-ECD system following Paul *et al*.^34^. Animals were sacrificed by decapitation on the last day of treatment (84^th^ day) following 2 h of administration with salicylic acid (100 mg/kg; i.p). The cortex, striatum, hippocampus and substantia nigra regions of brain were either dissected out or micropunched, processed with ice-cold HClO_4_ (0.1 M) containing 0.01% EDTA and centrifuged at 10,000 × g for 5 min. The supernatant (10 μL) was injected into the HPLC-ECD system and the detection of 2,3- and 2,5-DHBA was performed at +740 mV against the standards. The composition of the mobile phase was 8.65 mM heptane sulfonic acid, 0.27 mM EDTA, 13% acetonitrile, 0.43% triethylamine and 0.22% orthophosphoric acid.

### Brain histology

The anesthetized animals (chloral hydrate; 350 mg/kg b.w.; i.p.) were perfused transcardially first with 30 ml ice-cold PBS (0.1 M; pH 7.4) followed by 50 ml of 4% paraformaldehyde (in PBS) for immunohistochemistry. Brains were removed from calvarium, kept overnight in the same fixative and cryoprotected in 30% sucrose. For histoenzymological studies, the anesthetized animals were perfused transcardially with 50 ml of ice-cold PBS, followed by 50 ml of 10% glycerol (in PBS). Brains were cryoprotected using 30% sucrose. Twenty micron thick coronal sections passing through the cortex, striatum, hippocampus and midbrain (mainly substantia nigra) regions of brain were made using Cryostat (0620E, Thermo Shandon, UK). Sections were collected on poly-L-lysine coated slides for histoenzymological studies and in well-plates containing PBS for immunohistological study^31,34^.

### Acetylcholinesterase histoenzymology

The sections from intended brain regions were washed in PBS and incubated in a green colored reaction mixture for 45 min at 37 °C. The reaction mixture (20 ml) composed of acetylthiocholine iodide (11 mg in 13 ml 0.1 M potassium phosphate buffer, pH 6.0), 1 ml of 0.1 M sodium citrate, 2 ml of 30 mM copper sulfate, 2 ml distilled water and 2 ml of 5 mM potassium ferricyanide^42^. Sections were then thoroughly washed with distilled water, mounted in DPX, and photographed using a digital SLR camera attached to Trinocular Microscope (Eclipse CiL, Nikon, Japan).

### Mitochondrial complex-II activity

Mitochondrial complex-II activity was analyzed according to Pandey *et al*.^41^ with minor modification^34^. The tissue sections from intended brain regions were activated by rinsing in PBS at 37 °C for 10 min and then incubated in the reaction mixture containing 0.03 mol/L NBT, 0.05 mol/L phosphate buffer (pH 7.4) and 0.05 mol/L sodium succinate at 37 °C in dark for 35 min. Sections were then washed in PBS, mounted in glycerol and photographed immediately under bright-field illumination using a digital SLR camera.

### Mitochondrial complex-III activity

Mitochondrial complex-III histochemistry was performed following Govindaiah *et al*.^43^ with minor modification^34^. The sections were rinsed in PBS and incubated in the reaction mixture at 37 °C for 50 min. Reaction mixture contained DAB (5.67 mg), cytochrome c (2.33 mg) and sucrose (0.5 g) in 10 ml PBS (0.1 M, pH 7.4). After incubation, the sections were rinsed gently with PBS, dried, mounted in DPX and photographed.

### GFAP-immunohistochemistry

The sections were washed in Tris-HCl-buffered saline (TBS, 0.1 M; pH 7.4) and incubated in 3% (v/v) H_2_O_2_ for 5 min. Blocking was done with TBS containing 10% donkey serum and 0.3% Triton X-100 for 1 h. Sections were incubated with rabbit polyclonal anti-GFAP antibody (1:700) diluted in TBS containing 2% donkey serum and 0.3% Triton X-100 for overnight at 4 °C. Then the sections were washed with TBS and were incubated with HRP-conjugated secondary antibody (1:1000) diluted in TBS containing 2% donkey serum and 0.3% Triton X-100 for 1 h. Colour was developed with the DAB-liquid substrate solution for 3 min and then the sections were rinsed thoroughly, dehydrated in increasing grades of ethanol, cleared in xylene, mounted in DPX and photographed under bright-field illumination^31^.

### Nitric oxide synthase (NOS) histoenzymology

NOS histoenzymology in brain tissues was preformed following Madathil *et al*.^44^. Coronal sections were incubated in the dark at 37 °C for 60 min with the reaction mixture (0.5 M Tris–HCl buffer, 1.5 mM β-NADPH, 0.25 mM NBT, 0.25% Triton X-100, and 15 mM CaCl_2_). Sections were then washed in PBS, dried, mounted in glycerol and photographed.

### Densitometric analysis

The intensity of the characteristic colour of mitochondrial complex-II and III was measured using Fiji Version of ImageJ software by calculating the optical density^34,45^. Optical density was calculated from serial sections from five different brain samples of each group of mice. Optical density is the logarithm of maximum intensity by mean intensity, where theoretical maximum intensity is 255.

### Statistical analysis

Statistical analysis was performed employing an unpaired Student’s *t*-test (two-tailed) and a two-way, repeated measures ANOVA with factor ‘intervention’ (diet) and ‘brain regions’ (cortex, striatum, hippocampus and substantia nigra) using the software GraphPad Prism version 7.0 for Windows. Results are given as mean ± S.E.M. Values of *P* ≤ 0.05 were considered significant.

## Results

### Hypercholesterolemia causes cognitive impairment

**Figure 2:**
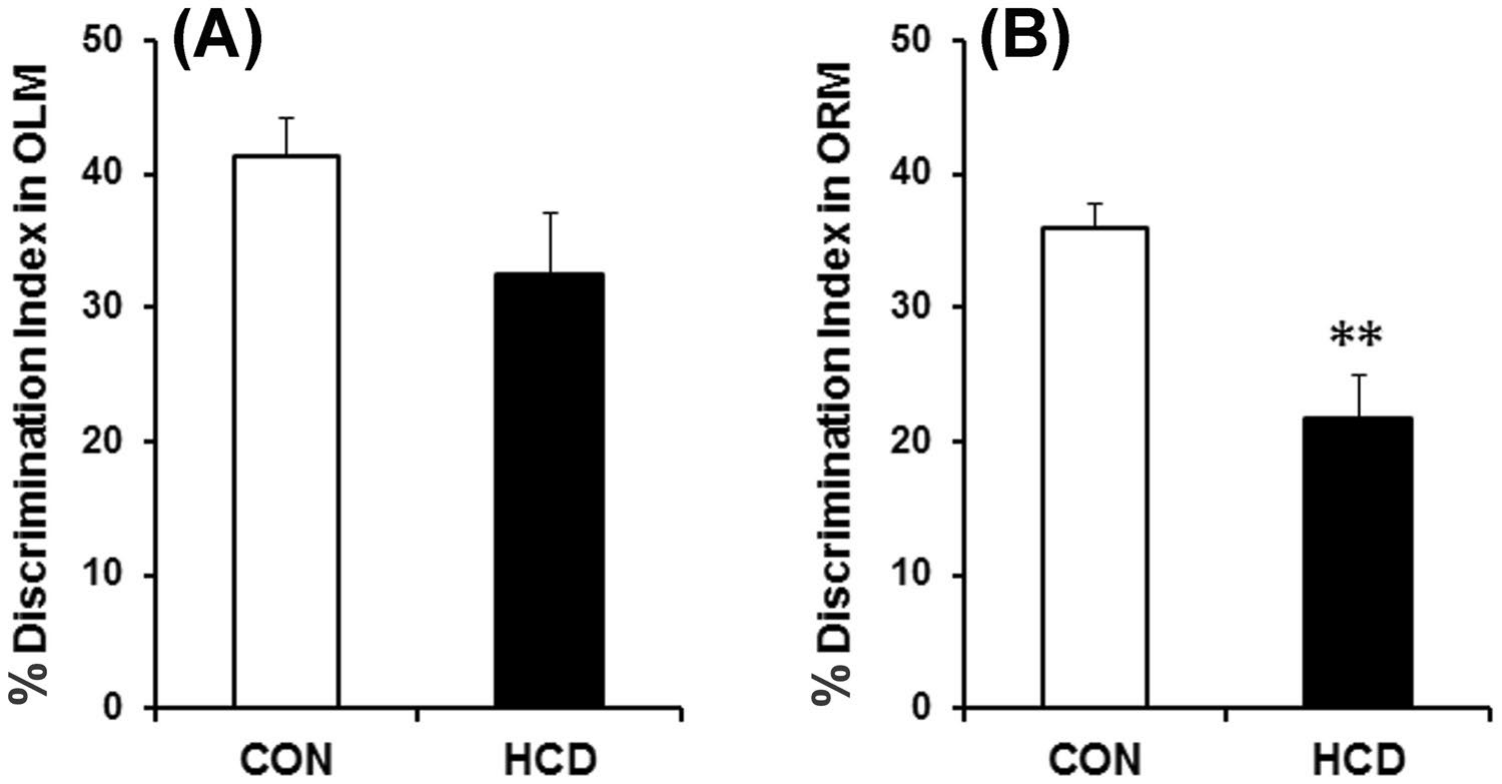
Hypercholesterolemia on cognitive functions in mice. **(A)** Short-term Object Location Memory (OLM; 90 min) and (**B**) Long-term Object Recognition Memory (ORM; 24 h). DI was calculated as: [(time exploring the novel object − time exploring the familiar)/(time exploring novel + familiar) × 100]. The results are mean ± S.E.M. **P ≤ 0.01 as compared to control. Data were analyzed using an unpaired Student’s t-test.

The object location and object recognition tasks are the most reliable and widely used tests to evaluate cognitive performance, which involve animal’s innate preference for novelty and are not stressful^32^. Short-term memory was tested in OLM as there was 90 min interval between training and test, while ORM was used for long-term memory as the test was performed 24 h following training^32^. During training sessions of OLM and ORM, there occurred no significant differences in total number and time of exploration in both the objects in high-cholesterol diet fed mice compared to the control (data not shown). During OLM and ORM test, the high-cholesterol diet fed mice give less preference to the object placed in the novel location or novel object respectively compared to the control. From the time of exploration between the objects in OLM and ORM tests, DI was calculated. The DI was decreased by 9% in OLM test in high-cholesterol diet fed group which was however did not differ significantly compared to the normal diet fed control animals (Fig. 2A). In ORM test, the DI was found to be decreased significantly by 15% in animals those were subjected to high-cholesterol diet as compared to the control (Fig. 2B). Thus, high-cholesterol diet group of mice exhibited significantly retarded long-term memory compared to the control animals which was evident from the DI score of ORM tests.

### Hypercholesterolemia increases brain cholesterol content

**Figure 3:**
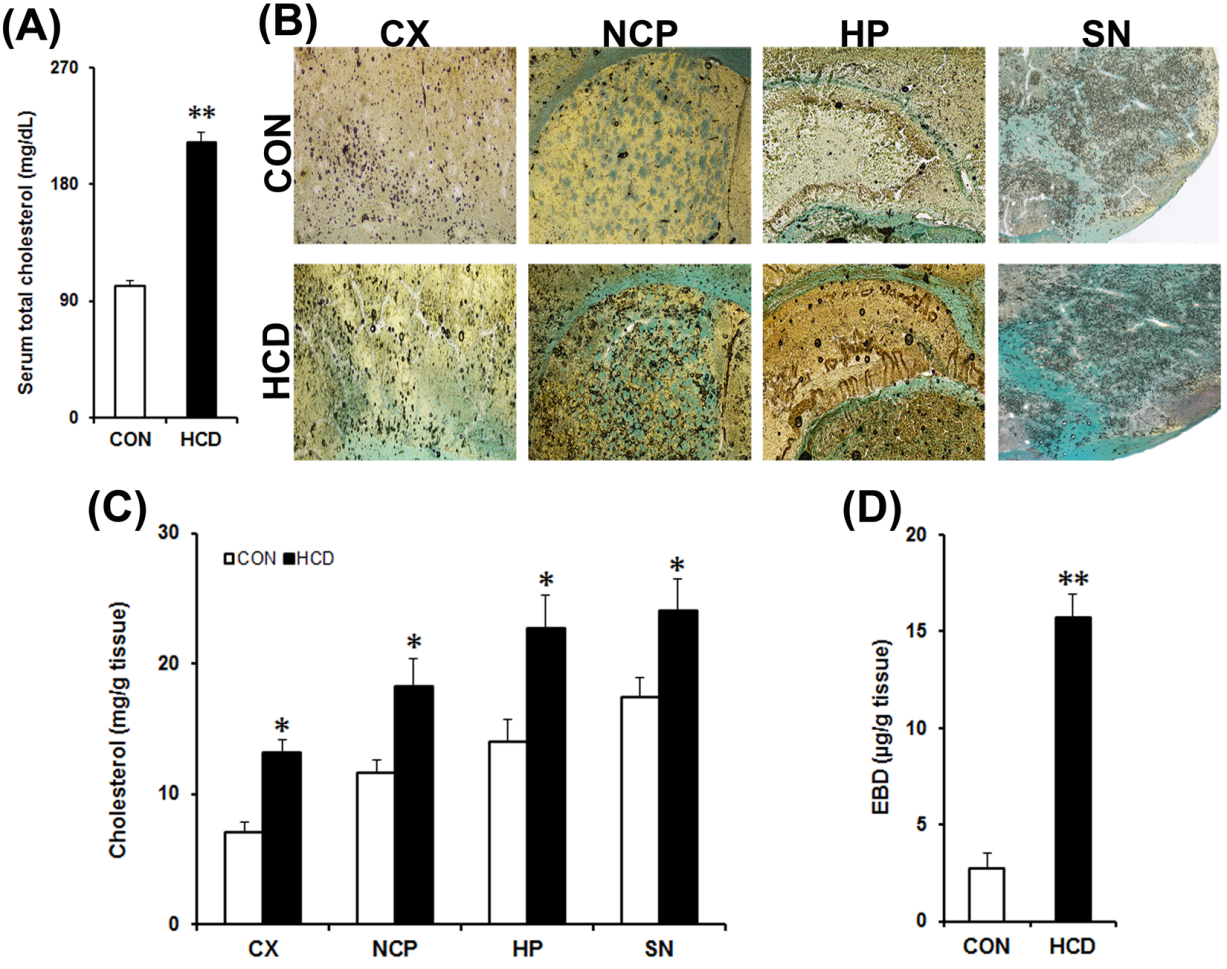
High-cholesterol diet causes hypercholesterolemia, increases cholesterol level in brain and disrupts blood-brain barrier (BBB). Control (CON) mice received normal diet and the HCD group received high-cholesterol diet (at 5% w/w mixed with normal diet) for 12 weeks and sacrificed on the last day. (**A**) Cholesterol level in blood serum (n = 8). (**B**) Cholesterol level in brain. Brain cholesterol level was assayed histologically in paraformaldehyde fixed tissue sections following Schultz’s method. Photographs are representative sections showing the cortex (CX), striatum (NCP), hippocampus (HP) and substantia nigra (SN) from control and HCD mice. The blue or greenish-blue colour denotes cholesterol. The dark circles denote air bubbles formed as a result of acid (sulphuric acid: acetic acid) reaction. Photographs were taken at 4× magnification. (**C**) Estimation of brain cholesterol level. Cholesterol level was estimated from tissue homogenates of CX, NCP, HP and SN regions of brain by using kit (n = 6). (**D**) BBB disruption. Evans Blue dye (EBD) extraversion in brain tissues were analysed for possible disruption of BBB using a Spectrophotometric method (n = 4). The results are mean ± S.E.M. *P ≤ 0.05 or **P ≤ 0.01 as compared to control. Data of cholesterol level in serum (**A**) and brain EBD were analyzed using an unpaired Student’s t-test (**D**), and brain cholesterol levels were analyzed using a two-way repeated measure ANOVA (**C**).

Hypercholesterolemia is the elevated levels of cholesterol in blood^46^. Mice that were subjected to high-cholesterol diet for 12 weeks had significantly elevated levels of total cholesterol in blood compared to the normal diet fed control mice (Fig. 3A). The mean total cholesterol level in blood was found to be 101 ± 7.5 mg/dL and 208 ± 15.7 mg/dL in the control and high-cholesterol diet group respectively. Thus, high-cholesterol diet for 12 weeks caused a 2-fold (P = 0.003) increase in blood cholesterol level, which is consistent with our previous mice model of hypercholesterolemia^31,34^.

Cholesterol level in brain was estimated to ascertain the effect of hypercholesterolemia on the level of cholesterol in brain using histochemistry and spectrophotometric quantification methods. The cholesterol in tissues appears blue or greenish-blue due to its oxidation in air with iron alum, followed by treatment with acetic-sulphuric acid mixture, the intensity of which signifies the level of cholesterol^35^. Hypercholesterolemia in mice caused a visible accumulation of cholesterol in different regions of brain (Fig. 3B). Based on the integrity of tissues, we have standardized the time that showed the highest intensity of blue-green colour following exposure of tissues with the acid mixture for taking the photograph in each area. The standardized time was found to be 5 min for cortex and striatum, 3 min for hippocampus and 1 min for substantia nigra.

A spectrophotometric method was used to quantify the levels of cholesterol in discrete brain regions. There occurred a significant increase in the level of cholesterol in the cortex, striatum, hippocampus and substantia nigra regions of brain of hypercholesterolemic mice compared to the control (Fig. 3C). In hypercholesterolemic animal cholesterol level was increased significantly by 1.85-fold in cortex (P = 0.019; 13.16 ± 1.02 *vs*. 7.09 ± 0.8 mg/g), 1.56-fold in striatum (P = 0.024; 18.23 ± 2.2 *vs*. 11.67 ± 0.97 mg/g), 1.62-fold in hippocampus (P = 0.016; 22.77 ± 2.5 *vs*. 14.01 ± 0.1.7 mg/g) and 1.5-fold in substantia nigra (P = 0.043; 24.1 ± 2.43 *vs*. 17.45 ± 1.54 mg/g) as compared to the control.

### Hypercholesterolemia causes disruption of BBB

Extravasation of albumin-bound EBD into brain tissue was estimated to ascertain disruption of BBB. Animals were injected with 2% EBD (4 ml/kg b.w.) on the last day of diet and sacrificed 22 h post injection. The content of EBD, measured in whole brain lysate, was found to be significantly higher by 6-fold (P = 0.002) in brain of hypercholesterolemic mice compared to the control (15.7 ± 1.2 *vs*. 2.7 ± 0.81 µg/g; Fig. 3D). A higher quantity of EBD in the brain indicates disruption of the BBB^41^.

### Hypercholesterolemia causes decrease in brain AChE activity

**Figure 4:**
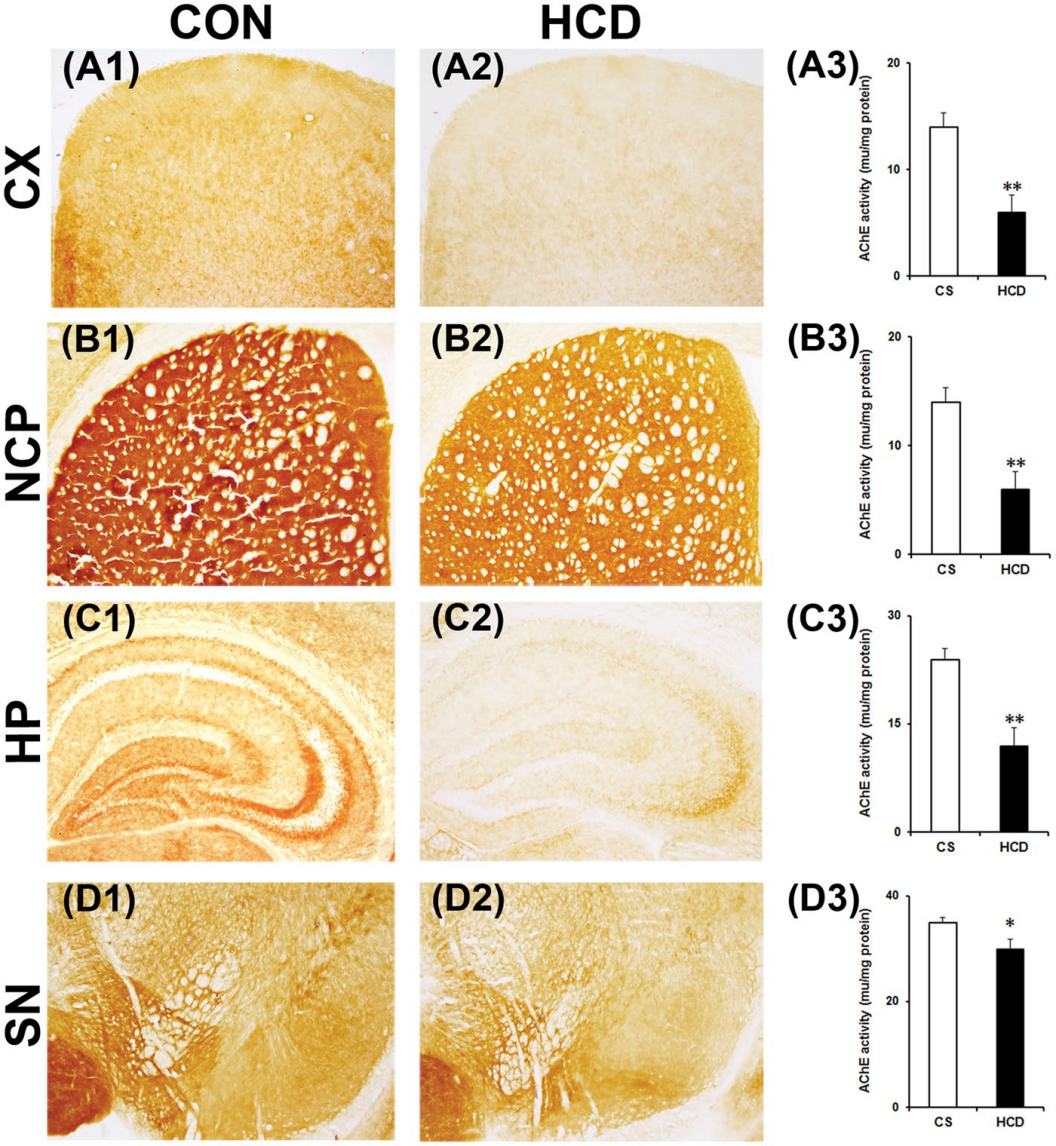
Hypercholesterolemia reduces acetylcholinesterase (AChE) activity in brain. Animals were sacrificed and perfused with 10% glycerol on the last day of 12 weeks on normal diet (control; CON/CS) or high-cholesterol diet (HCD). 20 µm coronal serial sections of different brain regions were processed for AChE histoenzymology. Photographs are representative sections of AChE staining showing the cortex (CX; A1 and A2), striatum (NCP; B1 and B2), hippocampus (HP; C1 and C2) and substantia nigra (SN; D1 and D2) of CON and HCD mice. Photographs were taken at 4× magnification. Activity of AChE in CX (A3), NCP (B3), HP (C3) and SN (D3) were quantified using Ellman’s method. Intended brain regions were dissected out and processed for the estimation of AChE activity by taking absorbency at 412 nm (n = 6). AChE activity decreased significantly in all the regions. Data represent AChE activity in mu/mg protein; and mean ± SEM. *P ≤ 0.05 and **P ≤ 0.01 as compared to CON of the respective regions of brain. P-values were calculated using a two-way repeated measure ANOVA.

AChE activity was assayed in coronal sections of different brain regions by a histoenzymological method using acetylthiocholine iodide as a substrate of the enzyme^42^. The result ensured a marked visible decrease in colour intensity in the cortex (Fig. 4A2), striatum (Fig. 4B2), hippocampus (Fig. 4C2) and substantia nigra (Fig. 4D2) regions of brain of hypercholesterolemic mice compared to the corresponding brain regions of the control mice (Fig. 4A1,B1,C1,D1). The characteristic brown color developed as a result of the reaction between copper ions and ferrocyanide (ferrocyanide is produced by reduction of ferricyanide by thiocholine, which is liberated following degradation of acetylthiocholine iodide by AChE). The decrease in colour intensity is an indicative of reduced enzyme activity^42^.

Ellman’s method was employed to estimate the activity of AChE in tissue homogenates of the intended brain regions using Spectrophotometer^36^. AChE activity was found to be reduced significantly by 57% in cortex (P = 0.003; Fig. 4A3), 31% in striatum (P = 0.008; Fig. 4B3), 50% in hippocampus (P = 0.005; Fig. 4C3), and 29% in substantia nigra (P = 0.039; Fig. 4D3) of hypercholesterolemic mice compared to control, which is similar to the histoenzymological results of AChE. Thus, the decrease in AChE activity in different brain regions has been confirmed by both the methods.

### Hypercholesterolemia reduces mitochondrial complexes activity with elevation in hydroxyl radical level in brain

**Figure 5:**
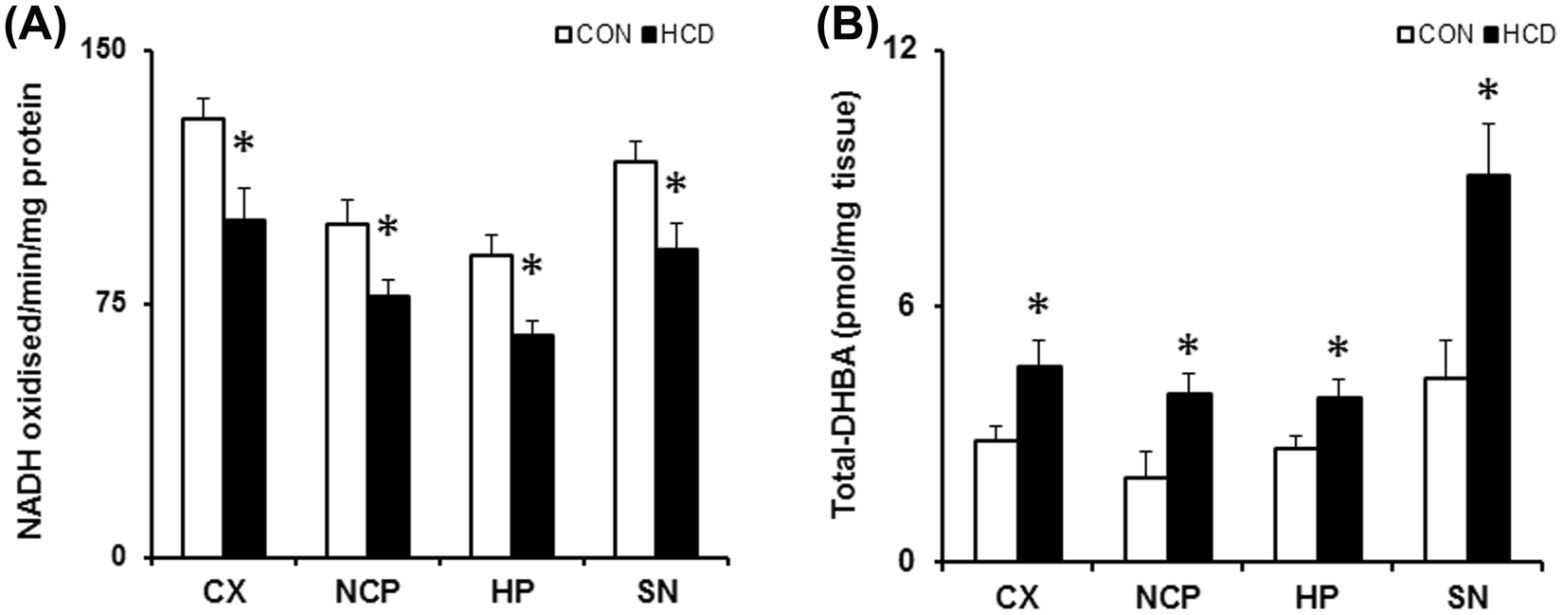
Hypercholesterolemia (**A**) diminishes mitochondrial complex-I activity and (**B**) elevates hydroxyl radical (•OH) level in brain. Mice were subjected to standard diet (control; CON) or high cholesterol diet (HCD) daily for 12 weeks. Animals were sacrificed by decapitation on the last day of treatment, different regions of brain, such as cortex (CX), striatum (NCP), hippocampus (HP) and substantia nigra (SN) were quickly dissected out and processed for specific activity assay of mitochondrial complex-I using NADH as a substrate and represented as nmol of NADH oxidized/min/mg protein (n = 6). For estimating the levels of •OH, animals were sacrificed 2 h post administration of salicylic acid (100 mg/kg; i.p.) on the last day of treatment. 2,3- and 2,5-dihydroxy benzoic acid (DHBA; •OH adducts of salicylate) formed were measured from tissue homogenates of intended brain regions using a sensitive HPLC-ECD method to measure the level of total DHBA. Data are expressed as pmol of DHBA per mg tissue. Data represented mean ± S.E.M. *P ≤ 0.05 as compared to CON (n = 6). A two-way repeated measures ANOVA was used to calculate P-values.

Mitochondrial complex-I activity was analyzed from crude mitochondrial fractions using NADH as the substrate on the last day of diet^40,41^. Hypercholesterolemia caused a significant reduction in activity of mitochondrial complex-I in all the intended brain regions. The complex-I activity (in amount of NADH oxidized/min/mg protein) was reduced by 23.08% (P = 0.021), 21.79% (P = 0.04), 26.66% (P = 0.017) and 22.34% (P = 0.041) respectively in cortex, striatum, hippocampus and substantia nigra in hypercholesterolemic mice compared to the control (Fig. 5A).

In mice, hypercholesterolemia caused a significant increase in the levels of •OH in different brain regions (Fig. 5B). The level of •OH was estimated by measuring the adducts of salicylic acid: 2,3- and 2,5-DHBA. In hypercholesterolemic animals, the levels of 2,3- and 2,5-DHBA were increased respectively by 2.17- and 1.54-fold in cortex, 1.5- and 2.03-fold in striatum, 1.54- and 1.44-fold in hippocampus, and 1.81- and 2.15-fold in substantia nigra as compared to control. Thus, the level of total DHBA (2,3-DHBA + 2,5-DHBA) was increased significantly by 1.61-, 1.98-, 1.45- and 2.09-fold in these brain regions of hypercholesterolemic mice compared to control (Fig. 5B).

**Figure 6:**
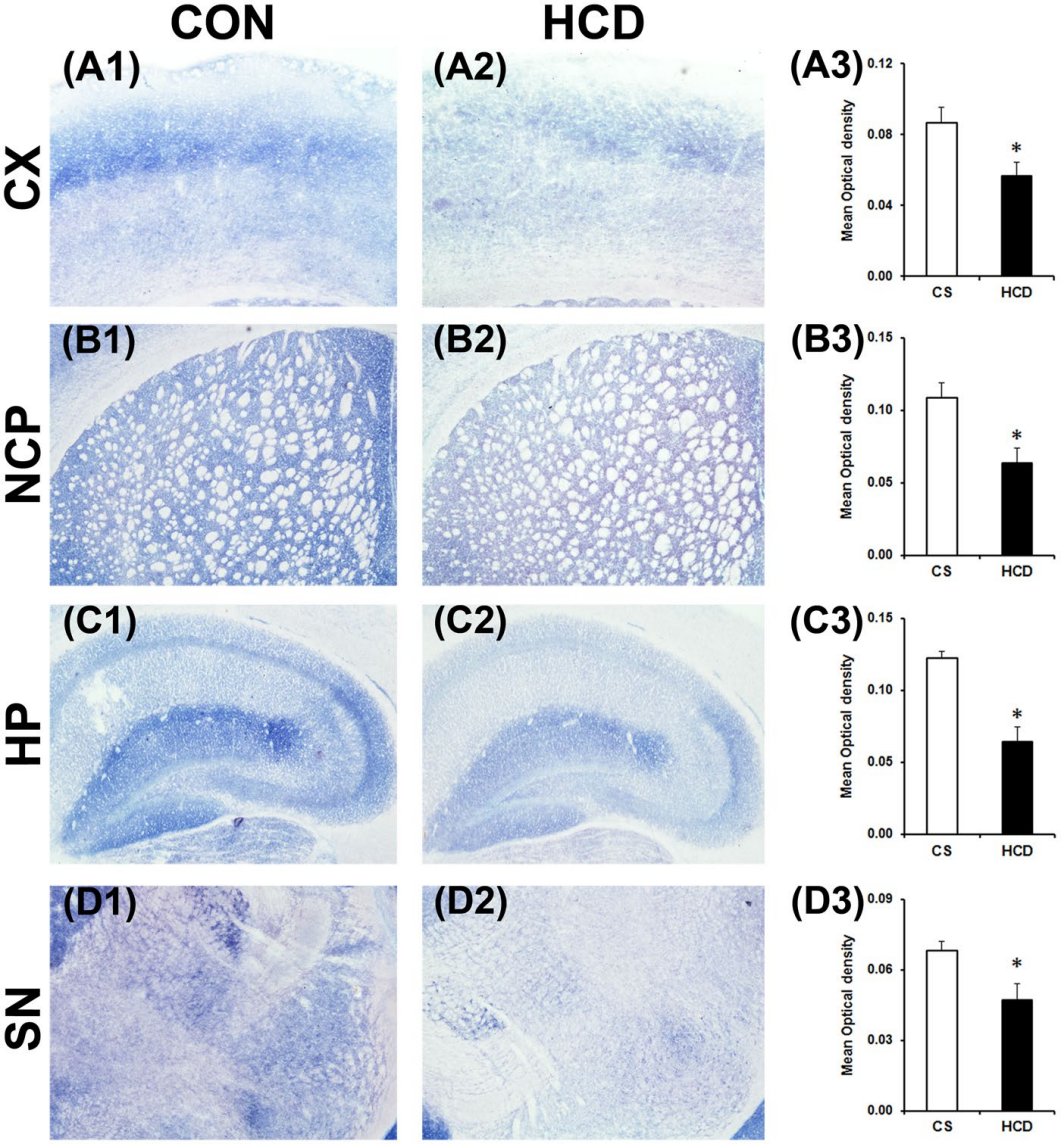
Hypercholesterolemia diminishes mitochondrial complex-II activity in brain. Representative photographs of histoenzymological staining of mitochondrial complex-II activity in cortex (CX; A1 and A2), striatum (NCP; B1and B2), hippocampus (HP; C1 and C2) and substantia nigra (SN; D1 and D2) regions of brain of control (CON/CS) and high-cholesterol diet (HCD) groups of mice. Photographs were taken at 4× magnification. Densitometric analysis of the photographs of complex-II staining (A3,B3,C3,D3). Photographs of four serial sections of CX (A3), NCP (B3), HP (C3) and SN (D3) of each group (n = 4) were analyzed using ImageJ software for determination of optical density. Data represented mean ± S.E.M. *P ≤ 0.05 as compared to CON. P-values were calculated using an unpaired Student’s t-test (two-tailed).

Activities of mitochondrial complex-II and complex-III were assayed using the substrates sodium succinate and cytochrome c respectively by histoenzymological method in the coronal sections of the intended brain regions. There occurred a marked visible decrease in colour intensity in all the regions of brain assayed for complex-II (Fig. 6), which signifies a decrease in the activity of the enzyme^34,41^. Densitometric analysis of the sections of complex–II activity assay revealed a significant decrease in activity of the enzyme in all the brain regions of hypercholesterolemic animals compared to the control. Complex–II activity was decreased significantly by 34.48% in cortex (P = 0.037; Fig. 6A3), 41.24% in striatum (P = 0.022; Fig. 6B3), 47.58% in hippocampus (P = 0.017; Fig. 6C3) and 29.98% in substantia nigra (P = 0.042; Fig. 6D3) of hypercholesterolemic animals.

**Figure 7:**
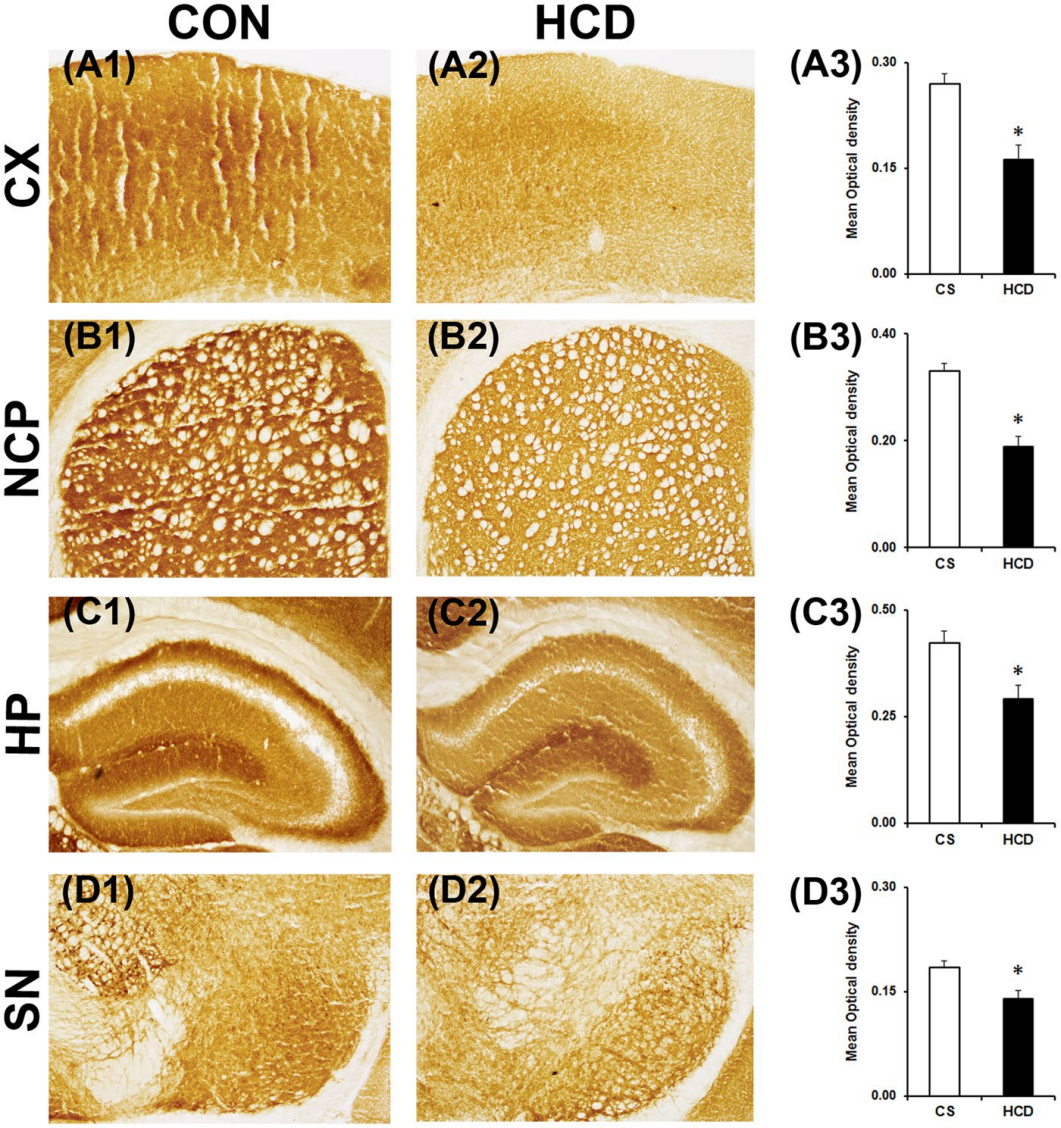
Hypercholesterolemia diminishes mitochondrial complex-III activity in brain. Representative photographs of histoenzymological staining of complex-III activity in cortex (CX; **A**1 and **A**2), striatum (NCP; B1 and B2), hippocampus (HP; **C**1 and **C**2) and substantia nigra (SN; **D**1 and **D**2) of control (CON/CS) and high-cholesterol diet (HCD) groups of mice. Photographs were taken at 4× magnification. Densitometric analysis of the photographs of complex-III staining (A3,B3,C3,D3). Optical density was analyzed from four serial sections of CX (A3), NCP (B3), HP (C3) and SN (D3) regions of brain of each group (n = 4). Data represented mean ± S.E.M. *P ≤ 0.05 as compared to CON. An unpaired Student’s t-test (two-tailed) was used to calculate P-values.

The histoenzymological analysis of mitochondrial complex-III revealed a visible decrease in colour intensity in all the brain regions of hypercholesterolemic mice (Fig. 7), which indicated a reduction in activity of the enzyme^34^. The activity of complex–III was decreased significantly by 40.04% (P = 0.018; Fig. 7A3), 43.08% (P = 0.026; Fig. 7B3), 31.13% (P = 0.043; Fig. 7C3) and 24.07% (P = 0.047; Fig. 7D3) respectively in the cortex, striatum, hippocampus and substantia nigra in hypercholesterolemic animals compared to the control.

### Hypercholesterolemia causes inflammation in brain

**Figure 8:**
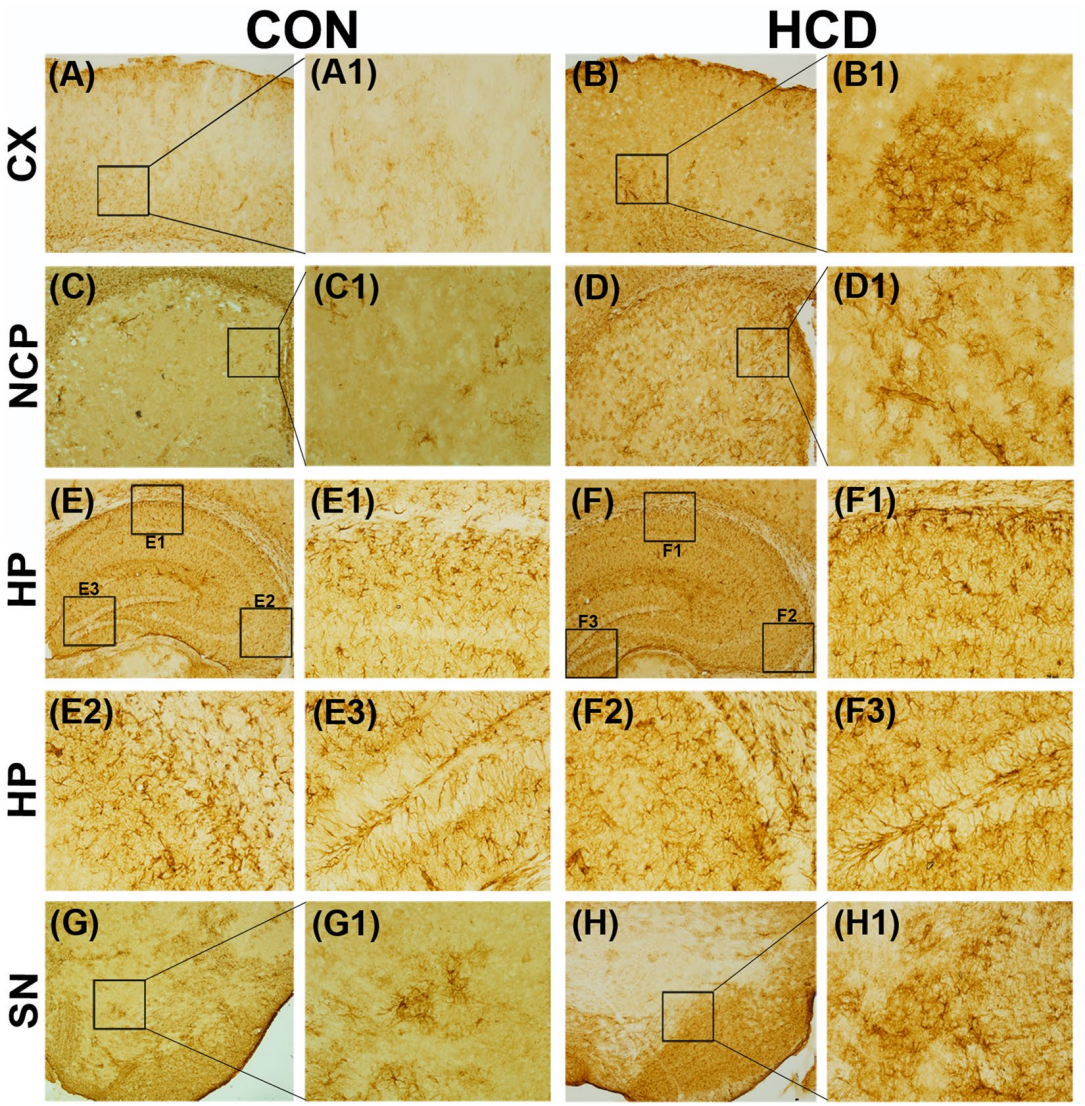
Hypercholesterolemia causes inflammation in glia. Animals were sacrificed and perfused with 4% PFA following 12 weeks (84^th^ day) on normal diet (control; CON) or high-cholesterol diet (HCD). The coronal sections passing through CX (**A**,**B**), NCP (**C**,**D**), HP CA1 (E1,F1), HP CA3 (E2,F2), HP DG (E3,F3) and SN (**G–H**) were processed for Glial fibrillary acidic protein (GFAP)-immunoreactivity. GFAP-reactivity was increased in all the brain regions of hypercholesterolemic mice (**B**,**B**1,**D**,**D**1,**F**,**F**1–**F**3,**H**,**H**1) compared to the corresponding brain regions of control mice (**A**,**A**1,**C**,**C**1,**E**,**E**1–**E**3,**G,G1**), which signifies astrocytosis due to inflammation. Photographs were taken at 4× and 20× magnification. CX = cortex; NCP = striatum; HP = hippocampus; SN = substantia nigra; CA = Cornus ammonis; DG = Dentate Gyrus.

**Figure 9:**
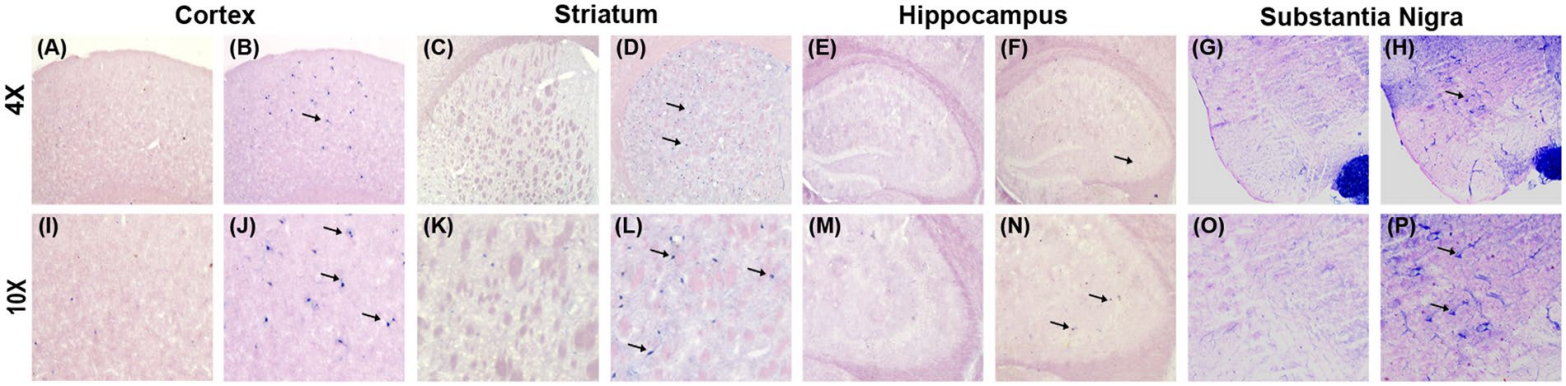
Hypercholesterolemia causes inflammation in neurons. Nitric oxide synthase (NOS) activity in cortex (**A**,**B**,**I**,**J**), striatum (**C**,**D**,**K**,**L**), hippocampus (**E**,**F**,**M**,**N**) and substantia nigra (**G**,**H**,**O**,**P**) regions of brain was analyzed by histoenzymology for possible inflammatory stress in neurons. NOS-active neurons were more in (**B**,**J**) cortex, (**D**,**L**) striatum, (**F**,**N**) hippocampus and (**H**,**P**) substantia nigra regions of hypercholesterolemic mice, compared to the corresponding brain regions of control mice (**A**,**C**,**E,G**). Arrows point to NOS-active neurons.

GFAP and NOS - the two markers of inflammation respectively in glia and neurons, were assayed in brain of hypercholesterolemic animals. GFAP expressed abundantly in reactive astrocytes under inflammatory stress and the process is known as astrogliosis^14,28^. GFAP-immunohistochemistry was employed to ascertain the effect of hypercholesterolemia on astrogliosis^31^. Marked visible increased in the number of GFAP-immunoreactive astrocytes were seen in the photographs of all the brain regions of hypercholesterolemic mice compared to control (Fig. 8). GFAP-positive astrocytes were counted using ImageJ software from the photographs of serial sections of different regions of brain^34,45^. There occurred significant increase in GFAP-positive astrocytes by 5-, 4.6-, 2.5- and 3.2- fold in the cortex, striatum, hippocampus and substantia nigra regions of brain respectively in hypercholesterolemic mice compared to the control (Supplementary Fig. 1). The number of NOSactive neurons was found to be higher in the brain regions, mainly in cortex, striatum and substantia nigra of hypercholesterolemic mice as compared to control (Fig. 9). Using ImageJ software the NOS-active neurons were counted from the photographs of serial section of different brain regions. NOS-active neurons were found to be increased significantly by 5-, 4- and 2.5-fold in the cortex, striatum, and substantia nigra regions respectively in hypercholesterolemic mice compared to the control (Supplementary Fig. 2).

## Discussion

Cholesterol homeostasis is vital for sustaining life since it is one of the pivotal constituents of cellular membrane and myelin sheath of neurons^47^. However, altered cholesterol homeostasis mostly due to its elevated levels in blood (hypercholesterolemia) has become a global health concern as it not only associated with cardiovascular diseases but also a prognostic risk factor for neurodegenerative disorders, particularly AD^30,46–49^. Recently, we have demonstrated that hypercholesterolemia in mice can cause Parkinson’s disease (PD)-like pathology and exaggerates the parkinsonian symptoms as well as pathologies in animal model of PD^30,31,34^.

The present finding that AChE activity is decreased in all the regions of brain of hypercholesterolemic mice (Fig. 4) is the most significant finding of the study and the first report of such kind, which provided an argument for its pathogenic influence to brain functions. Cholesterol-induced loss of AChE activity in brain is quite alarming since more than 5% of the adult Indian population and 0.5% globally are suffering from hypercholesterolemia^48,49^. Loss of brain AChE activity is evident in several brain disorders including neurodegenerative diseases^3–9^. Particularly in AD patients, the loss of AChE in brain regions, such as cortex and hippocampus is extensively reported^5–9^. Reduced AChE activity in the cortex of demented and non-demented PD patients^3^, as well as hippocampus of a rat model with hepatic encephalopathy, was reported earlier^4^.

Detrimental effect of hypercholesterolemia on cognitive functions of rodents and rabbits is known. Epidemiological studies and in animal models of hypercholesterolemia-induced by diet or by genetic manipulations reported to retard long-term or working memory^16–24^. Epidemiological studies suggest that high blood levels of cholesterol in midlife increases the risk of cognitive impairment^17^. Also, the patients with Familial hypercholesterolemic have displayed a high incidence of mild cognitive impairments^50^. Our result of long-term memory impairment by hypercholesterolemia is consistent with the earlier reports; however, our approach is novel in this model (Fig. 2B).

Our report on cognitive impairment and global loss of AChE activity in brain of hypercholesterolemic mice have resemblance with the with symptoms and pathology of AD^7–12,51,52^. However, the present finding is contradictory with previous reports on brain AChE activity in animal models of hypercholesterolemia^19,22,23,25^. Increased activity of AChE in cortex and hippocampus was reported in hypercholesterolemic Swiss albino mice that were given diets with 20% fat and 1.25% cholesterol for 8 weeks^23^. Although the study reported a significant increase in serum cholesterol, the cholesterol level was found much lesser compared to our hypercholesterolemic model^23^. Moreover, the present model is a chronic model of 12 weeks compared to 8 weeks model of Moreira *et al*.^23^. Rather, the mice were fed with high quantity of fat (20%) and thus, the increase in AChE activity might have been affected by fat and not cholesterol^53,54^. Thus, the differences in the results may be due to the time of exposure of cholesterol to the animals and composition of the diet.

Significantly elevated level of cholesterol in different brain regions of hypercholesterolemic mice (Fig. 3B,C) is one of the significant findings of the study. Interestingly, the regions of brain where AChE activity was most depleted (cortex and hippocampus; Fig. 4), the levels of cholesterol were also found to be higher in those regions (Fig. 3B,C). Since cholesterol homeostasis in the brain regulated through *de novo* synthesis, with limited import from the peripheral circulation^29,30,47^, to investigate the mechanism behind the increase in brain cholesterol level in hypercholesterolemic mice, BBB integrity was tested using Evans Blue dye extraversion assay^39^. The result demonstrated a higher quantity of the dye breaching the BBB (Fig. 3D), and thereby indicates that hypercholesterolemia causes BBB disruption^38,39^. Compromised BBB integrity has been reported in rodents subjected to high-cholesterol diet^55,56^, which has also been revealed in the present study. This might be the underlying cause of increased cholesterol level in brain in the hypercholesterolemic mice.

The finding highlighted the involvement of mitochondrial dysfunction (Figs 5A, 6,7) and inflammation (Figs 8,9) towards the diminished activity of brain AChE. Mitochondrial dysfunction at respiratory chain complexes and severe neuroinflammatory process has been implicated in several neurodegenerative diseases, including AD^57,58^. Inhibition of mitochondrial complexes (I and II) activity was reported in the cortex of genetic model of hypercholesterolemia^26,27^. Recently, we have demonstrated inhibition of mitochondrial complexes activity in dopamine-rich regions of brain subjected to cholesterol-rich diet^34^; however, present result on the loss of mitochondrial complexes activity globally in brain of hypercholesterolemic mice is the first report of such kind. Functional impairment of mitochondria due to inhibition of respiratory chain complexes is known to produce toxic free radicals, like hydroxyl radical, superoxide ions, which in turn cause mitochondrial dysfunction^59,60^. Therefore, the level hydroxyl radical in different regions of brain of the hypercholesterolemic mice was estimated. The result demonstrated elevated generation of hydroxyl radical in the brain regions of hypercholesterolemic mice where the activity of mitochondrial complexes was found to be reduced significantly (Fig. 5A,B). In our recent contribution, we have shown elevated generation of hydroxyl radical in midbrain dopamine-neurons of mice subjected to cholesterol-rich diet^34^.

Our result also provided evidence of induction of astrogliosis, an abnormal increase in the number of astrocytes^14,28,31^, represented by GFAP-immunoreactivity, comprehensively in brain of hypercholesterolemic mice (Fig. 8). Elevated number of astrocytes in a region represent cellular damage and underlying inflammatory stress^14,28,31^. In hypercholesterolemic animals, severe neuroinflammatory processes and associated gliosis mainly in the cortex and hippocampus regions of brain are known^14,16,28^. Astrocytes number is reported to increase in the places of damaged BBB^61^, which is also evident in the present study (Fig. 3D). NOS activation in neurons is known to hamper mitochondrial complexes function^36,46^. In neurons, NOS catalyzes the production of nitric oxide^62^. Although nitric oxide is an important neuronal messenger, however, it has the potential to inhibit mitochondrial complexes activity^44,63^.

Excess cholesterol in brain as well as in peripheral organs is metabolized to oxysterols which can easily traverse the BBB due to their polar nature^30,64^. Neurotoxic potentials of oxysterols are well-known and their implication in neurodegenerative diseases, such as AD and PD, are also evident^30,65^. The observed effects of hypercholesterolemia on brain whether mediated by the direct participation of cholesterol or indirect contribution via oxysterol is the limitation of the present study, and thus further study warrants in this direction.

The present study provided significant evidence of hypercholesterolemia-induced global loss of AChE activity with mitochondrial dysfunction and inflammation in discrete brain regions. Since the prevalence of hypercholesterolemia is alarmingly high and ever increasing, the present findings provided the evidence of involvement of hypercholesterolemia on brain dysfunction.

## Electronic supplementary material

Supplementary Information
