# Supplementary material for: Global loss of acetylcholinesterase activity with mitochondrial complexes inhibition and inflammation in brain of hypercholesterolemic mice
Source: Sci Rep. 2017 Dec 20;7:17922. doi: 10.1038/s41598-017-17911-z (PMC5738385; doi:10.1038/s41598-017-17911-z)
Supplement: Supplementary file 1 — Supplementary Information [file 41598_2017_17911_MOESM1_ESM.pdf]

Global loss of acetylcholinesterase activity with mitochondrial complexes inhibition and inflammation in brain of hypercholesterolemic mice.

Rajib Paul[1,2] & Anupom Borah[1]

[1]Cellular and Molecular Neurobiology Laboratory, Department of Life Science and Bioinformatics, Assam University, Silchar-788011, Assam, India.
[2]Department of Zoology, Pandit Deendayal Upadhyaya Adarsha Mahavidyalaya (PDUAM), Eraligool-788723, Karimganj, Assam, India.

Correspondence and requests for materials should be addressed to A. B. (anpom.borah@aus.ac.in)

Supplementary information

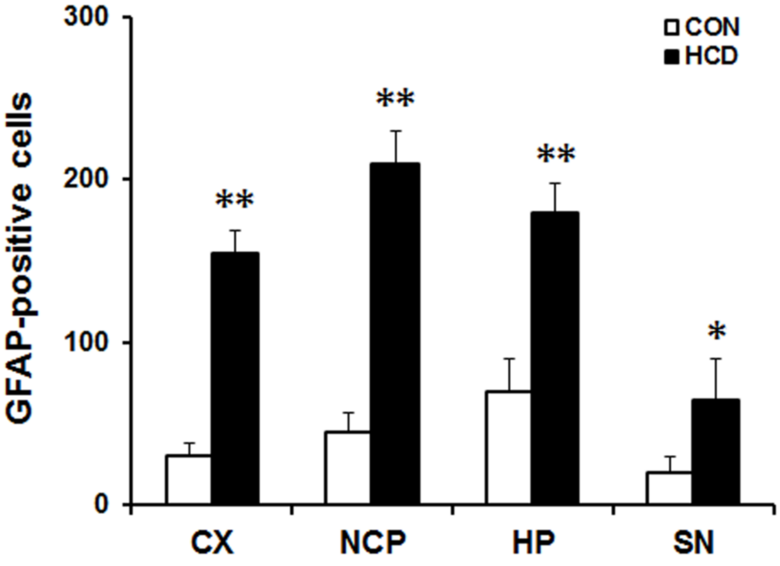

Figure S1. Counting of Glial fibrillary acidic protein (GFAP)-positive astrocytes in brain. PFA fixed serial cryo-sections (20 µ) of different regions of brain, viz. cortex (CX), striatum (NCP), hippocampus (HP) and substantia nigra (SN) from control (CON) and high-cholesterol diet (HCD) fed groups of mice were processed for GFAP-immunoreactivity. All the photographs were taken in same resolution and light intensity under bright field illumination in the same day. Photographs of serial sections of CX, NCP, HP and SN were taken from each group (n=5) to count the GFAP-positive astrocytes using Fiji version of

ImageJ software. The mean value of CA1, CA3 and DG regions from each photograph of hippocampus were taken. Results are expressed as Mean ± SEM. *$P \leq 0.05$ and **$P \leq 0.01$ as compared to CON of the respective regions of brain. P-values were calculated using an unpaired Student's t-test (two-tailed).

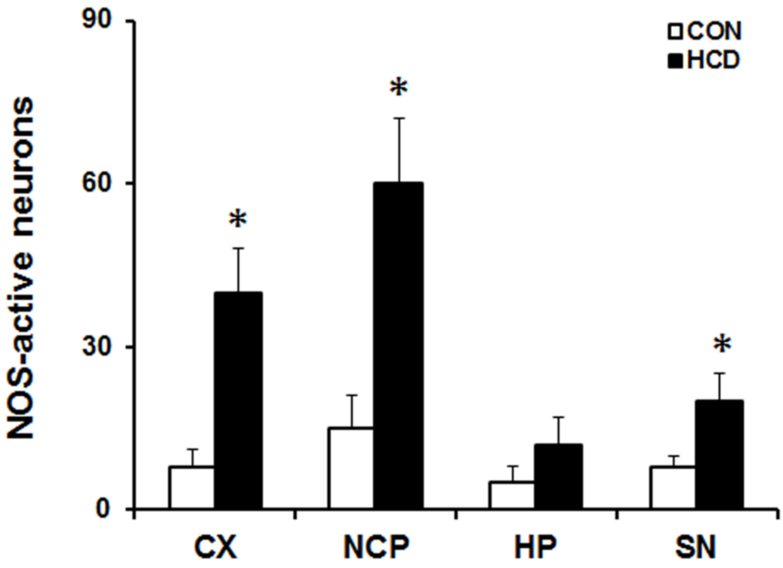

Figure S2. Counting of Nitric Oxide Synthase (NOS)-active neurons in brain. Glycerol perfused serial cryo-sections (20 µ) of different regions of brain, viz. cortex (CX), striatum (NCP), hippocampus (HP) and substantia nigra (SN) from control (CON) and high-cholesterol diet (HCD) fed groups of mice were processed for NOS-histoenzymology using NBT as the colouring agent. All the photographs were taken in same resolution and light intensity under bright field illumination in the same day. Photographs of serial sections of CX, NCP, HP and SN were taken from CON and HCD group of mice (n=5) to count the NOS-active neurons using ImageJ software. Results are expressed as Mean ± SEM. *$P \leq 0.05$ as compared to CON of the respective regions of brain. P-values were calculated using an unpaired Student's t-test (two-tailed).
